# Poly-tobacco use and mental health in South Korean adolescents

**DOI:** 10.18332/tid/187077

**Published:** 2024-05-22

**Authors:** Min Kwon, Eunjeong Nam, Jinhwa Lee

**Affiliations:** 1 Department of Nursing, The University of Suwon, Hwaseong-si, Republic of Korea; 2 Department of Nursing, Seoul Women's College of Nursing, Seoul, Republic of Korea; 3 Department of Nursing, University of Ulsan, Ulsan, Republic of Korea

**Keywords:** smoking, adolescents, tobacco use, mental health, poly-tobacco use

## Abstract

**INTRODUCTION:**

With the advent of new tobacco products, poly-tobacco use among adolescents is increasing. Smoking among adolescents negatively impacts both their physical and mental health. This study aimed to determine poly-tobacco use among adolescents in South Korea and to identify the mental health problems caused by single-, dual-, and poly-tobacco use.

**METHODS:**

Data from 54948 adolescents in the 2020 Korea Youth Behavior Web-based Survey were included. Mental health variables of our primary outcome were loneliness, anxiety, and depression. Descriptive statistics, Rao-Scott χ^2^ test and complex sample multivariable logistic regression analysis were conducted to determine the association between the type of tobacco product use and mental health.

**RESULTS:**

Among the subjects, 95.2% were non-tobacco users, followed by single (3.0%), dual (1.1%), and poly users (0.7%). The subjects with poly-tobacco use had significantly higher rates of loneliness (33.2%, p<0.001), anxiety (22.3%, p<0.001), and depression (49.9%, p<0.001) than those who used fewer tobacco products. Subjects who used poly-tobacco products were 2.13 (95% CI: 1.61–2.83) times more likely to report loneliness, 1.52 (95% CI: 1.12–2.07) times more likely to report anxiety, and 2.18 (95% CI: 1.68–2.82) times more likely to report depression than non-tobacco users.

**CONCLUSIONS:**

Among adolescents, poly-tobacco use is associated with symptoms of loneliness, depression, and anxiety, which are internalized mental health problems. Poly-tobacco use warrants early assessment of high-risk groups, education on the need for tobacco-use cessation, and active intervention for the psychological difficulties that these high-risk groups experience.

## INTRODUCTION

The emergence of new tobacco products provides several readily available opportunities for adolescent tobacco users. Poly-products use involving several types of tobacco products is emerging as an important public health issue. Compared to conventional cigarettes (CCs), new forms of tobacco products have a lower risk of exposing others in the surrounding environment and are often used for bonding with peer groups because of their attractive scent or trends^1^. Adolescents often try various new tobacco products because they have been incorrectly informed that these products are helpful in the process of quitting smoking or that they are less harmful than CCs^2^.

In March 2022, the US Food and Drug Administration (FDA) and the Centers for Disease Control and Prevention (CDC) released findings on the use of tobacco from the 2021 National Youth Tobacco Survey (NYTS)^3^. In 2021, approximately 2.55 million (9.3%) students reported current (past 30 days) use of a tobacco product. E-cigarettes (ECs) were the most commonly used tobacco product, cited by 2.06 million (7.6%) middle and high school students, followed by CCs (410000; 1.5%), cigars (380000; 1.4%), and smokeless tobacco (240000; 0.9%). The emergence of new tobacco products such as ECs in the US market has shifted the landscape of tobacco use among adolescents in the last decade toward poly-products use, of which ECs are a prominent component^4^. In 2020, the most common combination of products used by students in the US state of Minnesota who had used more than one product type in the past 30 days was ECs and CCs (28.3%). The second most common combination was ECs and cigars (19.9%), and the third most common was CCs, cigars, and ECs (10.2%)^5^.

In Korea, according to the 2019 Korea Youth Risk Behavior Web-based Survey (KYRBWS), 53.1% of smokers reported single-tobacco use, 24.8% reported dual-tobacco use, and 22.1% reported poly-tobacco use^6^. From 2016 to 2018, although there was no significant change in the rate of CC smoking among adolescent male students (from 9.64% to 9.39%), poly-tobacco use increased from 2.94% to 3.32%, and the proportion of poly-tobacco use among smokers in that population increased from 30.4% to 35.4%^7^.

The physical harm associated with new tobacco products has been reported, as has the difficulty in ceasing the use of these products because their nicotine content has been increased. In adolescents, the symptoms of nicotine dependence due to poly-tobacco use are more severe than those from regular CC use. Adolescent smokers who use multiple tobacco products have more symptoms of tobacco dependence, such as cravings and urges to smoke, and dual users have shown 4.46 times higher nicotine dependence than CC users^8^. Furthermore, it has been reported that dual users have shown a 0.56 times lower intention to quit smoking within 30 days, hindering quitting. These problems eventually can lead to dual- and poly-tobacco use^8^. According to previous studies, the additional use of new cigarette products, including dual- and poly-tobacco use, was shown to be associated with binge drinking and drug use. Its association with mental health problems, such as stress, loneliness, depression, anxiety, and suicidal tendencies, has also been reported^9^.

The mental health of adolescents is integrally related to their growth and development. Thus, the scope of the problem is larger and more complex than that of adults. Throughout the life cycle of human development, and during adolescence in particular, mental health has an important influence^10^. It is a definitive period for healthy growth and behavior throughout life. Among adolescent mental health factors, internalized symptoms – such as depression, anxiety, and loneliness – are the most frequently cited problems^11^.

This study aimed to determine the poly-tobacco use among adolescents and to identify the mental health problems associated with single-, dual-, and poly-tobacco use. Based on previous studies, we hypothesized that these three types of tobacco use have a greater influence on depression, anxiety, and loneliness compared to absence of tobacco use. Therefore, this study aimed to provide basic data on adolescent mental health and health-related behavior by identifying the association of poly-tobacco use among adolescents with depression, anxiety, and loneliness.

## METHODS

### Study samples and data sources

This is a secondary dataset analysis of data from the 16th (2020) Korean Youth Risk Behavior Web-based Survey (KYRBWS). The KYRBWS is an annual nationwide cross-sectional survey conducted by the Ministry of Education, the Ministry of Health and Welfare, and the Korean Centers for Disease Control and Prevention (KCDC). The Steering Committee, the Coordination Advisory Committee, and the Advisory Board Subcommittee are operated to efficiently conduct surveys and review questionnaires and the data^12^.

The study examines the health-risk behaviors of Korean adolescents (aged 12–18 years).

Representative students from middle schools and high schools in Korea were selected using stratified and multi-stage clustered probability sampling methods and asked to answer the validated questionnaires. The KCDC Institutional Review Board approved the procedures for the KYRBWS. Informed consent from the participants was obtained.

We used a sample of 54948 respondents from 800 schools (400 middle schools and 400 high schools) in the 2019 survey, with a response rate of 94.9%. Approval from the University of Ulsan was obtained on 27 August 2020 (IRB No.1040968-E-2022-002).

### Measures

*Variables*

Based on the existing literature^13^, the variables in this study were gender, grade in school, academic performance, co-residence with family, perceived economic status, secondhand smoke, current drinking, current smoking, perceived stress, physical activity, subjective health, and body mass index (BMI, kg/m^2^). Academic performance was reclassified into three categories: low, middle, and high. Co-residence with family was recoded as yes (living with family) or no (not living with the family, but living in a boarding house, dormitory, or foster home, living with a relative, or living alone). Perceived economic status was recoded as low, middle, or high. Secondhand smoke exposure at home and school was assessed with the following items: ‘How many days per week are you exposed to tobacco smoke at home?’, and ‘How many days per week are you exposed to tobacco smoke in school when indoors in a non-smoking area (classroom, toilet, hallway, etc.)?’. The responses were dichotomized: yes, for responses of one to seven days, and no. Current drinking was assessed with the following item: ‘On how many of the past 30 days did you have more than one drink?’. The responses were dichotomized as yes for responses of one day to every day, and no. Current smoking was assessed with the following item: ‘On how many of the past 30 days did you use CC, EC, or heated tobacco products (HTP), even one puff?’. If an adolescent used any of these products, regardless of the number of days, he or she was considered as currently using tobacco. Based on the answers, we classified the participants into three groups of single, dual, and poly users. Adolescents who used CC only, EC only, or HTP only were considered single users. Dual users were those who used both CCs and ECs, CCs and HTPs, or ECs and HTPs. Poly users were adolescents who used all three types of products. Perceived stress was reclassified into two categories: low (never, little, or a little) and high (high or very high). Physical activity was assessed with the following item: ‘On how many of the previous seven days did you engage in physical activities in which your heart rate increased or you were out of breath (regardless of type) for more than 60 minutes?’. The responses were dichotomized as yes for one day to seven days, and no. Subjective health was classified into two categories: healthy (very healthy or healthy) and unhealthy (average, unhealthy, or very unhealthy). BMI was calculated from height and weight, and was classified into four categories (underweight, normal, overweight, obese) using age- and sex-specific cut-off points.

*Mental health*

Mental health variables included loneliness, anxiety, and depression^8,11^. Loneliness was assessed with the following item: ‘How often have you felt lonely, in the past 12 months?’. The responses were reclassified into two categories: yes (very often or often) and no (sometimes, rarely, or never). Anxiety was measured using the Generalized Anxiety Disorder (GAD-7) scale. GAD-7 has the advantage of effectively screening for anxiety disorders in a short time and can also evaluate the severity of anxiety symptoms and functional decline, making it widely used in primary care^14^. It is seven-item questions answered with a four-point Likert scale ranging from 0 to 3. The total scores ranged from 0 to 21. When screening for an anxiety disorder, a cut-off point of 10 or greater is recommended^15^. In the present study, those scoring 10 or higher were classified as having an anxiety disorder. Depression was assessed with the following item: ‘In the past 12 months, have you felt sad or hopeless enough to shut down your daily routine for two weeks?’. The responses were dichotomized as yes and no.

### Data analysis

The data were analyzed using IBM SPSS Statistics version 27. Two-tailed p values <0.05 were considered statistically significant. The general characteristics and mental health of the subjects were analyzed using frequency and percentage. A Rao-Scott χ^2^ test was conducted to determine the bivariate relationship of mental health by type of tobacco products. Association between the type of tobacco product use and mental health was evaluated by complex sample multivariable logistic regression in which adjusted odds ratio (AOR) and 95% confidence intervals (CIs) were calculated, adjusting for control variables. Based on a previous study13, we included gender, grade in school, academic performance, co-residence with family, perceived economic status, secondhand smoke, current drinking, current smoking, perceived stress, physical activity, subjective health, and body mass index (BMI) as control variables.

## RESULTS

### General characteristics of the subjects

Table 1 summarizes the general characteristics of the subjects. Approximately 51.9% of the subjects were male and 50.4% were high school students. Approximately 37% of the subjects reported high academic performance. Most subjects resided with their family (96.2%). Fewer than half (47.5%) perceived their socioeconomic status as middle class.

**Table 1:** General characteristics of subjects (N=54948)

*Characteristics*	*n*	*%*
**Gender**		
Female	26595	48.1
Male	28353	51.9
**School**		
Middle	28961	49.6
High	25987	50.4
**Academic performance**		
High	20146	36.9
Middle	16585	30.1
Low	18217	33.0
**Co-residence with family**		
Yes	52332	96.2
No	2616	3.8
**Perceived socioeconomic status**		
High	21339	39.9
Middle	26397	47.5
Low	7212	12.6
**Type of tobacco product**		
None	52260	95.2
Single	1640	3.0
Dual	671	1.1
Poly	377	0.7
**Secondhand smoke**		
No	38564	70.5
Yes	16384	29.5
**Currently drinks alcohol**		
No	49056	89.3
Yes	5892	10.7
**Currently smokes**		
No	52260	95.2
Yes	2688	4.8
**Perceived stress**		
Low	36286	65.8
High	18662	34.2
**Physical activity**		
No	46817	86.0
Yes	8131	14.0
**Subjective health status**		
Healthy	38444	69.6
Unhealthy	16504	30.4
**BMI**		
Underweight	3595	6.9
Normal	37099	69.5
Overweight	3719	6.8
Obese	9121	16.8

n: unweighted. %: weighted. BMI: body mass index (kg/m^2^)

Among the subjects, 95.2% were not tobacco product users, followed by single (3.0%), dual (1.1%), and poly (0.7%) users. Approximately 30% of the subjects had experienced secondhand smoke. Overall, 10.7% were current drinkers and 4.8% were current smokers. Of the subjects, 34.2% reported that they perceived a high level of stress. Nearly 14% reported that they engaged in physical activity. Approximately 70% of the subjects reported that they viewed themselves as healthy. Overall, 69.5% had a normal BMI.

### Mental health of the subjects

Table 2 outlines the mental health status of the subjects. Approximately 14.1% of the subjects reported that they felt lonely, 11.2% had anxiety, and 25.2% experienced depression.

**Table 2:** Mental health of subjects (N=54948)

	*n*	*%*
**Loneliness**		
No	47182	85.9
Yes	7766	14.1
**Anxiety**		
No	48849	88.8
Yes	6099	11.2
**Depression**		
No	41108	74.8
Yes	13840	25.2

n: unweighted. %: weighted.

### Bivariate associations between type of tobacco product use and loneliness, anxiety, and depression

Table 3 shows the bivariate relationship of the subjects’ mental health status and the number of tobacco product used. The subjects with poly-tobacco use had significantly higher rates of loneliness (33.2%, p<0.001), anxiety (22.3%, p<0.001), and depression (49.9%, p<0.001) than those who used fewer tobacco products.

**Table 3:** Bivariate associations between type of tobacco product use and loneliness, anxiety, and depression (N=54948)

*Type of tobacco product*	*Loneliness*		*Anxiety*		*Depression*	
	*n (%)*	*Rao-Scott χ^2^ p*	*n (%)*	*Rao-Scott χ^2^ p*	*n (%)*	*Rao-Scott χ^2^ p*
None	7073 (13.5)	328.341 <0.001	5630 (10.9)	119.717 <0.001	12654 (24.3)	515.896 <0.001
Single	400 (24.5)		276 (16.9)		689 (41.3)	
Dual	167 (24.4)		109 (15.9)		307 (45.8)	
Poly	126 (33.2)		84 (22.3)		190 (49.9)	

### Multivariate associations between the type of tobacco product use and loneliness, anxiety, and depression

Table 4 shows the multivariate association between the type of tobacco products and mental health. We adjusted for control variables that included gender, grade in school, academic performance, co-residence with family, perceived economic status, secondhand smoke, current drinking, current smoking, perceived stress, physical activity, subjective health, and BMI.

**Table 4:** Multivariate associations between type of tobacco product use and loneliness, anxiety, and depression (N=54948)

*Type of tobacco product*	*Loneliness*	*Anxiety*	*Depression*
	*AOR (95% CI)*	*AOR (95% CI)*	*AOR (95% CI)*
None (Ref.)	1	1	1
Single	1.44 (1.25–1.65)***	1.13 (0.96–1.34)	1.58 (1.39–1.79)***
Dual	1.34 (1.09–1.66)**	1.02 (0.81–1.28)	1.81 (1.51–2.17)***
Poly	2.13 (1.61–2.83) ***	1.52 (1.12–2.07)**	2.18 (1.68–2.82)***

AOR: adjusted odds ratio; multivariable logistic regression analysis with adjustment for gender, grade in school, academic performance, co-residence with family, perceived economic status, secondhand smoke, current drinking, current smoking, perceived stress, physical activity, subjective health, and BMI.

** p<0.01,

*** p<0.001.

**Figure 1:**
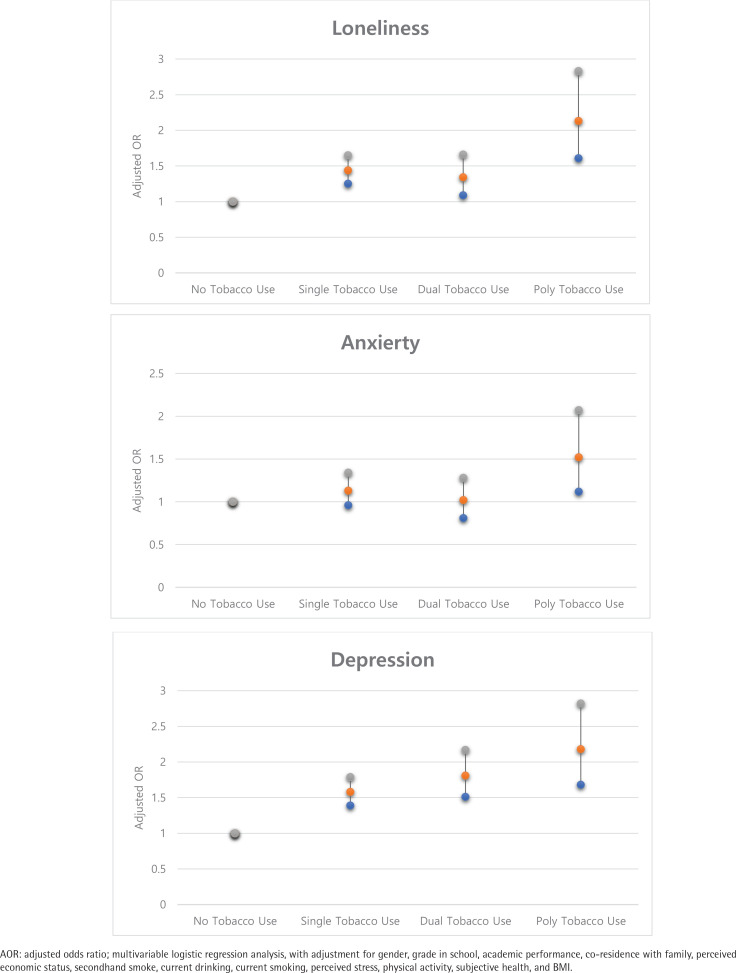
Adjusted odds ratios for loneliness, anxiety, and depression, according to tobacco use compared to no tobacco use

Using poly-tobacco products was associated with a two-fold increase in the odds of experiencing loneliness (AOR=2.13; 95% CI: 1.61–2.83). Subjects who used poly-tobacco products were 1.52 times more likely to report anxiety than non-smokers (AOR=1.52; 95% CI: 1.12–2.07). Subjects who used poly-tobacco products were 2.18 times more likely to report depression than non-smokers (AOR=2.18; 95% CI: 1.68–2.82) (Figure 1).

## DISCUSSION

This study determined the association between the type of tobacco products used and mental health, among Korean adolescents. The results indicate that single-, dual-, and poly-tobacco users are more likely to experience mental health problems compared to non-smokers, with poly-tobacco use being particularly associated with higher reporting of mental health problems.

First, regarding the relationship between smoking and loneliness, this study confirmed that adolescents who consumed tobacco, whether they were single-, dual-, or poly-tobacco users, experienced 1.44, 1.34, and 2.13 times more frequent loneliness than non-smokers, respectively. According to a previous study^16^, adolescent loneliness was 1.42 times more often associated with tobacco use compared to adolescents who did not currently use tobacco. Loneliness is commonly experienced during adolescence and has been associated with negative health outcomes^16^. Adolescents start smoking in the hope of building or strengthening social relationships^17^. In the period when their peer relationships are most important, undesirable behaviors occur through a drive for emotional belonging, and emotional support structure and friendship ties with others are important^18^. However, although lonely adolescents might try different types of cigarettes to attract friendships, this typically does not resolve their loneliness^19^. For this reason, smoking initiated to fit in with peer relationships eventually becomes the starting point for adolescents to be exposed to various tobacco products^17^, and such repetitive behavior makes adolescents even more lonely.

A systematic review^20^ conducted to confirm the relationship between smoking and loneliness did not explain why people complaining of loneliness smoke more tobacco. However, it presented the hypothesis that lonely individuals may smoke to enhance their social connections, or that the neuropharmacological effects of nicotine may engender loneliness^20^. Poly-tobacco use can indicate behavior beyond simple curiosity or social relationship desire.

Second, in this study, dual- and poly-tobacco use was associated with depression and anxiety in adolescents. This study confirmed that poly-tobacco users complained of depression 2.18 times more frequently and of anxiety 1.52 times more than nonsmokers. According to a recent systematic review^21^ on the relationship between substance use and mental health problems among adolescents and young adults in the US and Canada, adolescents who smoked were 1.65 times more likely to be depressed and 2.21 times more likely to be anxious than non-smokers. A previous study in Korea reported that adolescents who smoked were 1.27 times more likely to be depressed and 1.49 times more likely to be anxious^22^. Use of more than one type of tobacco signified more serious use. A previous study^23^ of Korean adolescents found that use of both ECs and CCs increased depression and suicidal thoughts. Similarly, the Population Assessment of Tobacco and Health (PATH) study^24^ confirmed the substance abuse and mental health problems associated with poly-tobacco use. The study classified very sad, depressed, and anxious as internalization problems, which were more frequent with poly-tobacco use than single tobacco use. Depression and anxiety were exacerbated by exposure to more numerous tobacco products.

Thus, poly-tobacco is closely associated with potential mental health issues during adolescence^9^. Compared to single users, dual users are exposed to higher levels of nicotine, and nicotine stimulation induced users to try more types of tobacco products^25^. Exposure to various types of tobacco, including those combined with smoking, results in frequent nicotine stimulation in adolescents^26^. This causes a craving for other forms of nicotine stimulation^25^. Tobacco use may act as a gateway to more serious drug use^27^. Therefore, an understanding and broad consideration of adolescent poly-tobacco users are necessary^9^.

In general, when it comes to mental health issues in adolescents, many previous studies have placed a great deal of emphasis on the importance of mental health during adolescence^28^ and discuss the need for appropriate interventions^29^. The adolescent brain is still in the formative stage, adapting to the many social, physical, sexual, and intellectual changes that occur during this period of development. It is also the time when most clinical onset of mental disorder arises. One in five adolescents has a mental illness that continues into adulthood^30^. This should be seriously taken when considering the advantage of timely interventions which increasing the chance of cure and reducing the cost of treatment.

Based on the results of this study, we make the following suggestions. First, to actively address adolescent smoking problems, it is recommended to conduct an early comprehensive assessment of their mental health. Adolescents may engage in poly-tobacco use as an expression of social relationships or resolution of psychological needs and are often unaware of the serious health consequences. In such a situation, the poly-tobacco user should be made aware of their consumed nicotine levels, the possible resultant changes in their bodies, and the potential health problems they may develop. Such notification can be a form of intervention. Additionally, it will be possible to confirm whether poly-tobacco use among adolescents leads to an increase in the number of tobacco products used or an increase in nicotine levels. Accordingly, this could provide approaches to the adolescent addiction problem^9^.

Second, it is necessary to limit the targeting of adolescents with new tobacco products. New tobacco products can be perceived as fashionable and attractive to young people who want to explore novel things^31^. The continuous search for new and interesting cigarettes causes poly-tobacco use, aggravating existing health problems compared to single tobacco use. Thus, exposure to new tobacco products should be prevented. Specifically, absolute restrictions on sales in youth sanctuaries, as well as restrictions on commercial advertisements and media exposure, can be considered^32,33^. Additionally, it may be considered to strengthen the promotion of tobacco harm^34^. In particular, considering improving the school climate could be beneficial, as it influences students’ tobacco behavior^35^.

### Limitations

This study has several limitations. We analyzed secondary data collected through a cross-sectional design and self-reporting; however, students are likely to conceal their smoking behaviors and may not accurately document them. Since less than 5% of Korean adolescents smoke, there are limits to generalizing this to the characteristics of all adolescents. In addition, it cannot be confirmed whether poly-tobacco use is associated with all variables related to mental health, and cross-sectional studies have limitations in explaining causality. And the inability to fully control for residual confounding poses limitations on the analysis.

Nevertheless, this study is significant because it links the increase in poly-tobacco use with mental health issues among adolescents in Korea, where school-centered health education related to adolescent smoking is currently underway.

## CONCLUSIONS

In this study, we found that the association between smoking and depression, anxiety, and loneliness among adolescents were greater in poly-tobacco users than in non-smokers. Compared to non-smokers, poly-tobacco users experience more frequent depression, anxiety, and loneliness. In particular, poly-tobacco use is related to negative health behaviors across adolescent mental health. For this reason, early assessment of mental health and intervention activities are needed to prevent adolescent smoking from leading to poly-tobacco use. It is recommended that particular high-risk groups be selected, objective biochemical indicators be implemented for them, and that they receive education about tobacco use to actively foster interventions. In addition, it is proposed that social devices and systems that can limit adolescents’ contact with new tobacco products be prepared. Accordingly, a systematic response is needed to prevent adolescent exposure to poly-tobacco use, which causes more frequent physical and mental problems than conventional smoking.

## Data Availability

The data supporting this research are available from the authors on reasonable request.
